# Graduate Study on the Continent—Germany

**Published:** 1908-06-27

**Authors:** 


### HOSPITAL ADMINISTRATION.

### CONSTRUCTION AND ECONOMICS.

### GRADUATE STUDY ON THE CONTINENT.—GERMANY.

#### VI.—THE PRIVATE CLINIC.

THE "private clinic," or rather the private polyclinic, is essentially a Continental institution, and it is one to which the graduate student would do well to pay some attention. In every town of Germany, for instance, that has any pretensions to being a centre for medical study, such institutions abound, and in many cases they have obtained a reputation which far outweighs that of the recognised polyclinics attached to the university or other public institutions. Thus at Berlin one of the best clinics is that of the celebrated specialist in nervous diesases, Dr. Oppenheim, who is not at present attached to the university, but whose private clinic is open to students. "A morning at Oppenheim's clinic is worth a day spent at the university clinic," has been said by men who are qualified to judge. These private polyclinics are generally near the large iustitutions. They are located in large flats, two or even more clinics being sometimes in the same building. On the outer wall, alongside the doorway, is a white enamelled plate, which bears in large black letters the name of the physician or surgeon in charge of the clinic, and the hours of attendance. The student who wishes to attend the clinic enters by a special door, which is reserved for "physicians and students," and is at once admitted to the consulting room, where he can introduce himself, by presenting his card or by writing his name in the visitors' book, to the head of the clinic. And here I may repeat what has already been stated, namely, that everywhere the student is received with the utmost courtesy and kindliness, and is afforded every opportunity for investigating and examining cases. This, of course, refers to the open polyclinic at which patients who fall into the category of "clubbers" are treated. In the private hospitals, which are really nursing homes for the treatment of in-patients, the student can only enter on the invitation of the physician or surgeon. Such invitations, however, are not exceptional, and where they are given the student often has a chance of learning much more than he would be able to learn in hospital wards or at out-patients'.

Closely related to these private polyclinics are the special hospitals, which agree, more or less, with the majority of our own charitable institutions for the sick. That is to say, they are not municipal or communal hospitals, like the majority of Continental hospitals, nor yet "Royal clinics" supported by the State, nor military lazarets under control of army medical departments, but they are institutions which are private in so far that they belong to, and are supported by, charitable organisations, receiving at the most, in a few exceptional cases, a moderate grant from the Government. They are governed by elected committees or boards, and are usually connected with a special denomination or body. In some cases their staffs are very fine, containing men who have a world-wide reputation, and the hospital consequently attracts a large number of students. At Berlin the best-known of these special hospitals are the Judische Krankenhaus (Hebrew Hospital), the Katholische Krankenhaus (St. Hedwig's Catholic Hospital), and the Bethanien Hospital, which is an institution of an evangelical sisterhood. At the first-mentioned hospital the surgical student will find one of the best operators, and one of the most original surgeons of Germany, Dr. Israels, whose work in the domain of urino-genital surgery is appreciated in England and America as widely as it is on the Continent. To the graduate student the fact that men like Dr. Israels and Dr. Oppenheim, both specialists whose names are known outside Germany, are not on the list of Berlin University professors is somewhat remarkable, but their exclusion is due to the rule which prevents the Hebrew from holding office under the Prussian Government.

At these special hospitals the graduate student is encouraged, and although there is no teaching in the ordinary sense of the word—that is to say, there are no regular classes for students and no systematic demonstration of cases—there is yet a good deal to be learned by watching methods. In this attention to methods, in comparing different ways of treatment, these special hospitals offer, perhaps, more scope than do the general clinics where, to a certain extent, one method prevails and stereotype regulations are the order of the day. At the special hospital the graduate is usually admitted everywhere upon production of his card.

The private polyclinic is open at various times, the most usual hour for the *Sprechstunde*, or consulting time, being 10—11 o'clock in the forenoon in the case of polyclinics and 1—3 in the afternoon in the case of clinics. To the former a much poorer class of patient comes, and a far greater variety of cases is to be seen. Usually there are interesting cases every day, but the percentages of "book cases" must of course vary, depending as they do on so many conditions. The visitor is admitted to the consulting room, and usually obtains a seat close to the chief of the clinic, who examines each case as it is brought in from the waiting room and briefly comments on it. Demonstrations are usually confined, except in those clinics which are much attended by junior men, to an explanation of interesting points, it being taken for granted that the visitors know all about the general theory of the case or its general pathology.

The student who desires to take a special course at a private clinic will find in most cases that his best way is to approach the chief of the clinic directly. In every case it is advisable that the student should attend, as a visitor, the clinical demonstrations at the particular clinic he wishes to attach himself to, so as to become acquainted with the professor. This allows of mutual personal study, and the student has a preliminary chance of familiarising himself with the peculiarities and methods of the man under whom he desires to serve. Then after a few such visits, he may broach the subject of a course. Generally the professors are chary of giving "single courses," though a student can obtain individual teaching if he pays for it. The ideal is, however, a small class, not exceeding five members. This distributes the fee charged more equally, and will be found in every case to work excellently, especially if (and this is very important in surgical work) the members arrange a programme beforehand and lay before the professor a definite scheme of work. The honorariums for such "private courses" are higher—usually much higher—than for courses at the regular clinics, and the question of relative value is, of course, one which each individual must decide for himself. As an average 150 marks per man for a daily course lasting for a month may be given; special courses, in cystoscopy, for instance, may run to 300 marks per man. Against this must be set the fact that the instruction given is very painstaking, full and clear, and that every opportunity is given to the student to do practical work.

